# Detecting Variable Resistance by Fluorescence Intensity Ratio Technology

**DOI:** 10.3390/s19102400

**Published:** 2019-05-26

**Authors:** Wanjun Sheng, Xiangfu Wang, Yong Tao, Xiaohong Yan

**Affiliations:** 1 College of Electronic and Optical Engineering & College of Microelectronics, Nanjing University of Posts and Telecommunications, Nanjing 210023, China; swjunz@163.com; 2 Key Laboratory of Radio Frequency and Micro-Nano Electronics of Jiangsu Province, Nanjing 210023, Jiangsu, China; 3 Nanjing RZisources International Trading Co., Ltd., Nanjing 210019, Jiangsu, China; wxfbyy@163.com

**Keywords:** resistance, reduced graphene oxide, fluorescence intensity ratio, sensitivity

## Abstract

We report a new method for detecting variable resistance during short time intervals by using an optical method. A novel variable-resistance sensor composed of up-conversion nanoparticles (NaYF_4_:Yb^3+^,Er^3+^) and reduced graphene oxide (RGO) is designed based on characteristics of a negative temperature coefficient (NTC) resistive element. The fluorescence intensity ratio (*FIR*) technology based on green and red emissions is used to detect variable resistance. Combining the Boltzmann distributing law with Steinhart–Hart equation, the *FIR* and relative sensitivity *S_R_* as a function of resistance can be defined. The maximum value of *S_R_* is 1.039 × 10^−3^/Ω. This work reports a new method for measuring variable resistance based on the experimental data from fluorescence spectrum.

## 1. Introduction

The resistor is a passive two-terminal electrical component with a major physical feature of converting electrical energy into thermal energy [1,2], which implements electrical resistance as a circuit element. The resistance value of an ideal resistive element whose current–voltage characteristic curve is linear is not changed with the voltage or current [3]. Nevertheless, a practical resistor in an electronic circuit exhibits non-linear behavior especially for some special resistive elements such as thermistors, varistors, and sensitive components [3,4,5,6]. Generally, the fixed resistance is easily measured. However, it is very hard to detect resistance with the high-frequency change in electronic devices during a short time interval. It is necessary to explore a new method to detect variable resistance during short time intervals.

Optical temperature sensing based on the fluorescence intensity ratios (*FIR*) has been reported as a fast-time response approach to detect the temperature in nanometer scale [7,8,9]. The relation between temperature and *FIR* can be obtained by fitting the *FIR* of thermally coupled levels at different temperature points [10,11,12]. The heat will be generated when the current flows through the resistance [13]. If this kind of heat can be detected by the *FIR* technology, and the relation between temperature and resistance can be determined, the variable resistance will be detected by the *FIR* technology. In this case, it is necessary to design composite materials that can emit luminescence as a resistive element.

**Figure 1:**
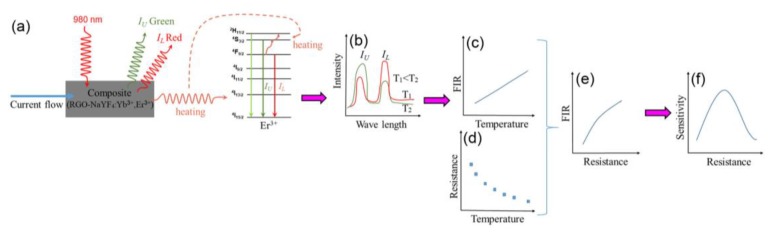
Schematic illustration of the mechanism of detecting variable resistance by fluorescence intensity ratios *(FIR)* technology. (**a**) Mechanism diagram of sensing under infrared excitation and current flow. (**b**) Fluorescence intensity. (**c**) The fitted curve of temperature dependent *FIR* based on Boltzmann distribution law. (**d**) Dependence of resistance on temperature. (**e**) The fitted curve of resistance dependent *FIR.* (**f**) Dependence of sensitivity on resistance.

**Figure 2:**
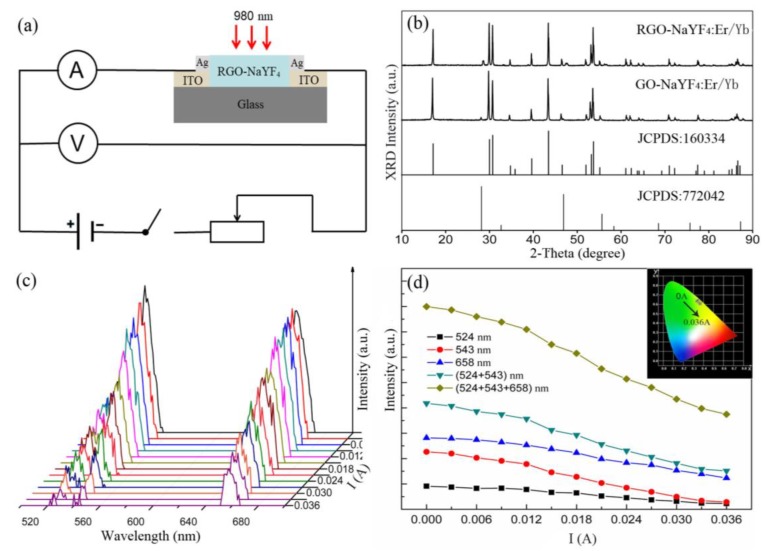
Experimental device and spectral measurement. (**a**) Schematic illustration of the experimental setup (including structures of variable-resistance sensor device). (**b**) The X-ray diffraction (XRD) patterns of composites GO-NaYF_4_:Yb^3+^,Er^3+^ and RGO-NaYF_4_:Yb^3+^,Er^3+^. (**c**) Current dependent luminescence spectra of the composite. (**d**) Fluorescence intensity of three up-conversion (UC), green and total emissions.

It has been widely reported that the lanthanide-ion doped NaYF_4_ with low phonon energy is the most commonly used optimal host material in up-conversion applications, since the nonradiative relaxation can be restrained efficiently [14,15]. Recently, the NaYF_4_:Yb^3+^,Er^3+^ fluoride nanomaterials have been reported for applications in biological imaging, solar cells, anti-counterfeiting, and optical sensors as multi-color phosphors [15,16,17,18,19,20]. It was found that reduced graphene oxide (RGO) with outstanding carrier mobility and high surface-to-volume ratio had been applied in material science, energy, and biomedicine [21,22]. The composite material composed of RGO and NaYF_4_:Yb^3+^,Er^3+^ will show bi-functional properties such as fluorescence and negative temperature coefficient (NTC) resistive element [23,24,25]. Since the fluorescence features of NaYF_4_:Yb^3+^,Er^3+^ are various, and the conductive characteristics of RGO can be affected by external disturbance of composite on account of its low density state surrounding the Dirac point [22], the resistance of composite material (RGO-NaYF_4_:Yb^3+^,Er^3+^) will be detected by using the spectrum property of NaYF_4_:Yb^3+^,Er^3+^ nanoparticles. In this paper, we design the composite nanomaterial (RGO-NaYF_4_:Yb^3+^,Er^3+^) as a variable-resistance sensor. The theoretical analysis process for detecting variable resistance by *FIR* technology is proposed, as shown in Figure 1. Since the current flowing the composite (RGO-NaYF_4_:Yb^3+^,Er^3+^) changes, the generated Joule heat changes and the adjacent energy levels (the upper level and lower level) of Er^3+^ can be thermally populated and depopulated in the photoluminescence process (Figure 1a). Therefore, the fluorescence intensity of I_U_ and I_L_ will change upon 980 nm excitation (Figure 1b). The *FIR* between I_U_ and I_L_ will change regularly and the temperature dependent *FIR* can be obtained according to Boltzmann distributing law [10,11,12], as shown in Figure 2c. The relation between temperature and resistance can be obtained from dependence of resistance on temperature according to Steinhart–Hart formula [23,24,25] in Figure 2d. Based on the above analysis, the *FIR* and relative sensitivity as a function of resistance can be derived, as shown in Figure 2e,f. Thus the variable resistance can be detected by *FIR* technology.

It is frequently reported to calibrate parameters such as humidity [26], strain and gas through calculating composites’ changing resistance [27,28,29]. However, the resistance is rarely measured by other parameters not to mention optical parameters. Compared with contact methods for traditional resistance measurement such as voltammetry, wheatstone bridge, and kelvin bridge [30,31], the *FIR* technology proposed in this study realizes non-contact survey and contributes to detecting fluctuating resistance significantly. Therefore, combining optical properties and measurement of resistance is of extreme significance and value, which contributes to expanding applications of optoelectronic devices. 

## 2. Materials and Methods

Starting materials included hydrochloric acid (AR), Er_2_O_3_ (99.99%), Yb_2_O_3_ (99.99%), Y_2_O_3_ (99.99%), NaF (AR), NaOH (AR), ethanol (AR), oleic acid (AR), and graphene oxide (GO). All the chemicals were used as received without any further purification. RGO-NaYF_4_:Yb^3+^,Er^3+^ composite was synthesized via a typical solvothermal procedure [32,33,34]. The Y_2_O_3_, Yb_2_O_3_, and Er_2_O_3_ were dissolved in hydrochloric acid and then heated to evaporate the water completely, which was dissolved in deionized water to obtain the stock solution of YCl_3_, YbCl_3_, and ErCl_3_ (0.5 mol/L). The homogeneous precursor solution was obtained through dissolving 210 mg of NaOH by 6.5 ml of deionized water, and adding sequentially 13 ml of absolute ethanol and 3.5 ml of oleic acid with continuous stirring for 15 min, then mixing consecutively 1.5 ml of 2 mg/ml GO dispersion solution and 1 ml of aqueous solution (0.5 mol/L) of 78% YCl_3_, 20% YbCl_3_, and 2% ErCl_3_ under agitation for 30 min. Subsequently, 252 mg of NaF was dissolved in 10 ml of deionized water, which was added into the above solution dropwise with vigorous stirring. Following agitating and then ultrasonicating for 30 min respectively to mix thoroughly. The mixed solution was transferred to a 100 ml of Teflon-lined autoclave, sealed, and heated at 200 °C for 10 h. Than the deposited product was collected after cooling to room temperature naturally, and gathered by consecutive washing with ethanol and centrifugation several times, then dried in air at 60 °C. In order to realize high conductivity of the composite, the conductive composite (RGO-NaYF_4_:Yb^3+^,Er^3+^) was achieved through heating under a nitrogen atmosphere at 700 °C for 30 min with heating and cooling rate of 5 °C/min.

The middle part of indium tin oxide (ITO) conductive glass substrate was etched to interrupted circuit, then cleaned with acetone and dried prior to use. The mixed solution of homogeneous dispersion was prepared by dispersing powder of the RGO-NaYF_4_:Yb^3+^,Er^3+^ composite into N-Methyl-2-pyrrolidone (NMP) solution and carrying out ultrasonication for 1 h. The composite sensing film on ITO glass substrate was achieved by drop coating the dispersion with micropipette, then annealed at 80 °C for 1h to remove the solvent. The two leads of copper wires were attached with silver paste on both sides of the film, and annealed at 90 °C for 30 min to obtain curing of electrical contacts. Thus, a resistance element as a sensor device was fabricated.

## 3. Results and Discussion

### 3.1. Experimental Device and Spectral Measurement

In order to study the performance quality of the variable-resistance sensor device, the measurement circuit is set up, as shown in Figure 2a. The sensor, irradiated by a fixed excitation source (980 nm), is connected to a series circuit composed solely of power supply, switch, sliding rheostat, voltmeter, and ammeter. The X-ray diffraction (XRD) patterns of GO-NaYF_4_:Yb^3+^,Er^3+^ and RGO-NaYF_4_:Yb^3+^,Er^3+^ as-prepared materials, show that the location and relative strengths of the diffraction peaks are capable of indexing to hexagonal (JCPDS Card no. 160334) and cubic (JCPDS Card no. 772042) mixed phase NaYF_4_ [35,36], as shown in Figure 2b. The up-conversion (UC) photoluminescence spectra of the sensor was measured with varying current under a fixed pump power (160.8 mW/mm^2^), among which, three emission bands were respectively ascribed to ^2^H_11/2_→^4^I_15/2_ (524 nm), ^4^S_3/2_→^4^I_15/2_ (543 nm), and ^4^F_9/2_→^4^I_15/2_ (658 nm) transitions of Er^3+^ ions [37,38,39], as depicted in Figure 2c. The luminescence intensity of three UC emission bands decreased with the increasing current, as described in Figure 2d. The CIE chromaticity in the inset of Figure 2d displays that the emission color transforms from green to greenish-yellow with the current intensity increasing. It means that the spectra and resistance of sensor are dependent on the current. In this process, the Joule heat will be generated.

### 3.2. Detecting Resistance Based on the FIR of 543 nm/524 nm

**Figure 3:**
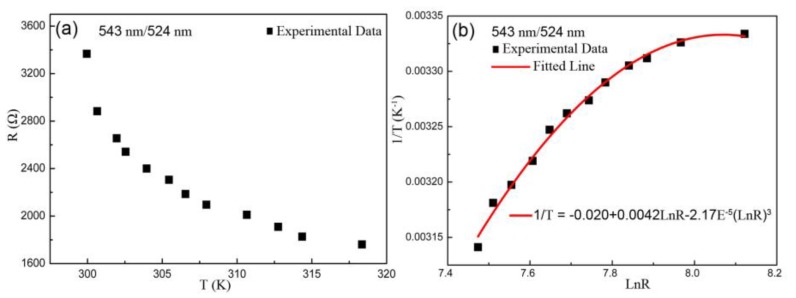
Plots of the ratio of 543 nm/524 nm including (**a**) the resistance–temperature experimental values, and (**b**) the fitted curve of resistance dependent temperature based on Steinhart–Hart formula.

Since the current produces Joule heat resulting in temperature variation [40], it can be observed that the resistance decreases monotonically with increasing temperature based on experimental data from Figure 3a. Specifically, the experimental values of temperature–resistance exhibited a typical NTC performance [24], which follows the empirical formula of thermistor named Steinhart-Hart equation:

(1) $$1/T=A+B(LnR)+C{\left(LnR\right)}^{3}$$

where *T* is in kelvin unit, *R* is the value of resistance, *A, B* and *C* are the fitted coefficients of curve. Based on the ratio of 543 nm/524 nm, the fitted curve of *LnR* dependent *1/T* obtained according to Equation (1) was in good agreement with experimental values, and the coefficients of the Steinhart–Hart equation were: *A* = −0.02, *B* = 0.0042, *C* = −2.17E^−5^, as shown in Figure 3b.

The voltage across the variable-resistance sensor device is alterable through adjusting the sliding rheostat at a fixed voltage in a circuit, which makes the current change (between 0 A and 0.036 A). The voltage affects the flow of the electrons through the resistance which affects the Joule heat generated in the resistance and results in temperature variation [41]. The *FIR* results from thermalization and it follows the Boltzmann distribution. The *FIR* of 543 nm/524 nm will be adjusted by the Joule heat. According to former studies [10,11,12], the *FIR* of two thermally coupled levels of ^4^S_3/2_ and ^2H^_11/2_ can be expressed as a function of *T* based on the Boltzmann distribution law as follow:

(2) $$FIR=Ae^{-\Delta E/KT}+B$$

where *A* and *B* are constants, *ΔE* is the energy difference between thermally coupled levels, *K* is the Boltzmann constant and *T* is the absolute temperature. 

**Figure 4:**
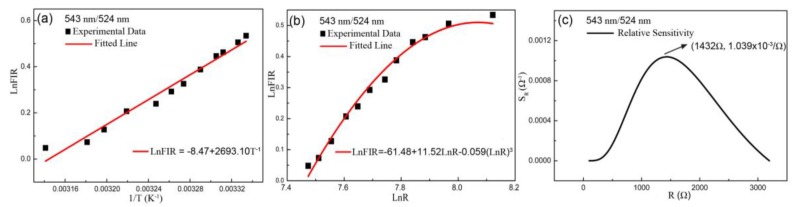
Plots of the ratio of 543 nm/524 nm including (**a**) the fitted curve of temperature-dependent *FIR* based on Boltzmann’s distribution law, (**b**) the fitted curve of resistance dependent *FIR*, and (**c**) the curve of resistance dependent relative sensitivity S_R_.

Equation (2) can also be rewritten as:

(3) LnFIR=−a/T+b

where *a* and *b* are fitted constants. The experimental points are scattered on or near the fitted linear curve of 1/*T* dependent *LnFIR* according to Equation (3), as shown in Figure 4a. One can find that the relation between the *LnFIR* and *1/T* tends to be linear. Combining Equations (1) and (3), the relation between the *FIR* and *R* can be calculated as the following formula:

(4) $$\begin{array}{ll} LnFIR & =-a(A+B(LnR)+C(LnR{)}^{3})+b\\ & =a_{0}+b_{0}(LnR)+c_{0}(LnR{)}^{3} \end{array}$$

where *a_0,_ b_0_* and *c_0_* are constants from fitted experimental data. Figure 4b describes that the experimental points can be well fitted by the formula in Equation (4) and *LnR* dependent *LnFIR* can be obtained. 

To estimate the performance quality of variable-resistance sensor, the relative sensitivity *S_R_* as a function of *R* is defined as:

(5) $$S_{R}=\frac{dFIR}{dR}=e^{a_{0}+b_{0}(LnR)+c_{0}{\left(LnR\right)}^{3}}[\frac{b_{0}}{R}+\frac{3c_{0}{\left(LnR\right)}^{2}}{R}]$$

Figure 4c displays the curve of *R* dependent sensitivity *S_R_*, which increases, then decreases with the increasing *R*, and arrives at maximum value of 1.039 × 10^−3^/Ω at 1432 Ω. The excellent sensitivity property of the sensor is apparent. 

### 3.3. Detecting Resistance Based on the FIR of 543 nm/658 nm

**Figure 5:**
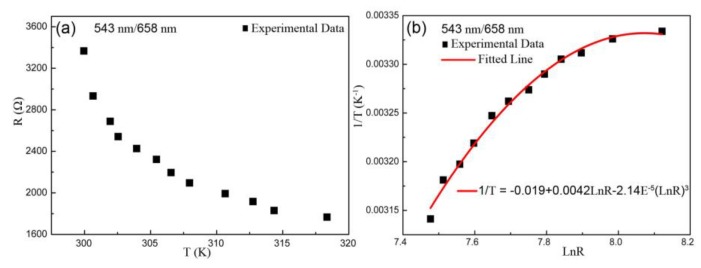
Plots of the ratio of 543 nm/658 nm including (**a**) the resistance–temperature experimental data, and (**b**) the fitted curve of resistance dependent temperature based on Steinhart–Hart formula.

**Figure 6:**
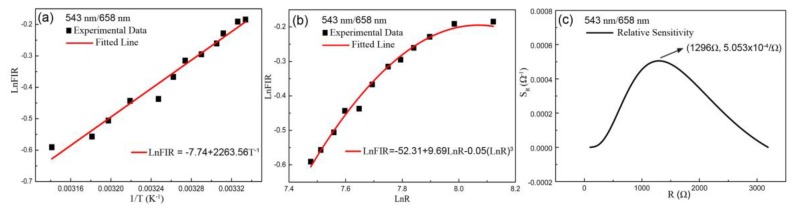
Plots of the ratio of 543 nm/658 nm including (**a**) the fitted curve of temperature-dependent *FIR* based on Boltzmann distribution law, (**b**) the fitted curve of resistance dependent *FIR*, (**c**) the relative sensitivity S_R_ as a function of resistance.

In the same theoretical analysis, it can be observed that the experimental values of temperature-resistance based on the ratio of 543 nm/658 nm still exhibits the characteristics of NTC resistance, as shown in Figure 5a. The curve of resistance-dependent temperature is fitted according to Steinhart–Hart equation in Figure 5b. The relation between *1/T* and *LnFIR* based on the *FIR* of 543 nm/658nm is linear according to Boltzmann’s distribution law, as drawn in Figure 6a. The fitted curve of *LnR* dependent *LnFIR* matched well with the experimental points, as shown in Figure 6b. And the *S_R_* maximum value of 5.053 × 10^−4^/Ω at 1296 Ω was obtained, as shown in Figure 6c. 

### 3.4. Sensitivity Stability of Sensor

**Figure 7:**
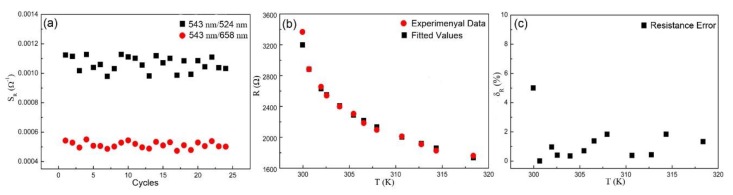
Sensitivity characterization of the sensor. (**a**) Dependence of relative sensitivity *S_R_* on the number of cycles. (**b**) The fitted and experimental data points showing the dependence of the resistance on temperature. (**c**) Dependence of the resistance error *δ_R_* on temperature.

Based on the electricity behaviors of the NTC resistance related to its composition, we expect the excellent luminescence property, reproducibility, and stability of variable-resistance sensor, in view of the chemical and luminescence characteristics of the conductive composite RGO-NaYF_4_:Yb^3+^,Er^3+^. To investigate the sensitivity stability of the sensor, the sensitivity data of multiple sets of cyclic measurements are described in Figure 7a. Under numerous cyclic measurements, the *S_R_* based on the *FIR* of 543 nm/658 nm is more stable and fluctuate less. The dependence of fitted and experimental data of resistance on temperature for sensor is shown in Figure 7b. The fitted values describing the evolution of the *R* as a function of the *T* almost matches with the experimental data. To study the effect of error, the fitted values of resistance based on Steinhart–Hart equation was added, which shows a slight difference compared to experimental data. Here, we define the relative error of the resistance as *δ_R_*:

(6) $$\delta_{R}=\frac{\left|R-R_{0}\right|}{R_{0}}\times 100\%$$

where *R_0_* is the experimental data and *R* is the fitted value. The temperature dependence of *δ_R_* is shown in Figure 7c. It can be observed that the resistance error *δ_R_* is comparatively small and stable. 

## 4. Conclusions

In summary, since the conductive composite RGO-NaYF_4_:Yb^3+^,Er^3+^ synthesized through the hydrothermal method exhibited characteristics of NTC resistance and fluorescence property, a late-model variable-resistance sensor device was designed. Since the voltage affects the flow of electrons through resistance, the Joule heat is generated in this process and the temperature is variable. The *LnR* dependent on *1/T* is investigated based on Steinhart–Hart equation. Furthermore, the *1/T* dependent *LnFIR* is studied based on Boltzmann’s distributing law. Therefore, the *FIR* and *S_R_* as a function of *R* can be attained using the *FIR* technology based on green and red emissions. The maximum *S_R_* value is 1.039 × 10^−3^/Ω at 1432 Ω based on the *FIR* of 543 nm/524 nm. The *S_R_* based on the *FIR* of 543 nm/658 nm exhibits more stable cycle performance. The resistance error *δ_R_* is comparatively small and stable. This work provides a novel strategy for measuring variable resistance by using an optical method during short time intervals.
